# Food Texture Acceptance, Sensory Sensitivity, and Food Neophobia in Children and Their Parents

**DOI:** 10.3390/foods10102327

**Published:** 2021-09-30

**Authors:** Maddalena Cappellotto, Annemarie Olsen

**Affiliations:** Section for Food Design and Consumer Behaviour, Department of Food Science, Faculty of Science, University of Copenhagen, Rolighedsvej 26, 1958 Frederiksberg C, Denmark; maddalenacappellotto@gmail.com

**Keywords:** texture, preferences, tactile, neophobia, children

## Abstract

This study aims to explore whether children’s food texture preferences are associated with different levels of sensory sensitivity and food neophobia, as well as with other variables, such as parental texture preferences. An online questionnaire was completed by 70 children aged 6–13 years old, alongside one of their parents. Generic texture preferences of children and parents were investigated with the Child Food Texture Preference Questionnaire (CFTPQ). Parents provided background information about their children by completing the Food Neophobia Scale (FNS), the Short Sensory Profile (SSP) and a Food Frequency Questionnaire (FFQ). The results showed that children who differed in their texture-liker status also differed in their levels of food neophobia and sensory information processing: children who preferred softer and non-particulate versions of foods were found to be more neophobic and sensory sensitive across all sensory domains. No relationship was found between parental and children’s texture preferences.

## 1. Introduction

Early school age is a sensitive time to support the development of healthy eating habits. To do so, it is essential to investigate the factors involved in food preferences, given the role they play in children’s food choices and intake, as well as in their growth and development [1,2,3]. Sensory properties were highlighted as one of the most influential factors determining eating behavior and within these, texture was shown to be a major reason for rejecting or accepting food in children [4,5,6].

Texture has been described as ‘the sensory and functional manifestation of the structural, mechanical, and surface properties of foods detected through the senses of vision, hearing, touch, and kinesthetics’ [7]. Certain texture attributes have been shown to elicit food rejection through disgust even before tasting in children [8]. Indeed, more than one sensory system may code aspects of food texture, and although most processing takes place in the mouth, an initial textural assessment can be performed visually before the food is ingested [9]. Even sounds that accompany food manipulation and breakdown can become signal elements of food texture and may influence its acceptance [9]. Across food products, hard, lumpy and granular textures are generally less accepted by children of all age groups [6,10,11,12]. However, a large European cross-cultural study recently showed that this trend may not be universal, but rather influenced by both cultural and individual differences [13]. These findings call for a further investigation of all possible factors contributing to the development of an individual’s texture acceptance and preferences. Among the aspects related to children’s characteristics, the degree of sensory sensitivity is one factor that may be of great importance in the development of texture preferences, as it was shown to be involved in rejection of certain textures [14].

Sensory sensitivity, also known as sensory over-reactivity, can be defined according to individual differences in the detection of, and reaction to, sensory information, including information from the taste, touch, vision, and smell senses [15]. Sensory sensitivity is believed to be an inherent characteristic [15], which has been associated with physiological markers. From a neurological point of view, individuals that are more sensory sensitive needs little sensory information to be able to register a stimuli, which can be conceptualized as having a lower threshold [16]. Moreover, the same individuals tend to manifest anxious tendencies that make them more receptive of changes in the sensory properties of food, which in turn could result in rejection [14]. Interestingly, higher levels of sensitivity to tactile and taste information were also related to food neophobia and pickiness [17], the first being defined as the reluctance to eat, or the avoidance of, new foods [2]. This behavior has been shown to be highly heritable [18,19] among omnivorous species, and it has been described as an evolutionarily beneficial survival mechanism to prevent children from ingesting noxious or toxic chemicals [20] once they become autonomous and mobile enough to reach foods from the environment without the supervision of parents or caregivers [21]. This phenomenon can be considered part of pickiness, due to which children accept a smaller variety of food items presented by their caregivers [1]. These age-related and temporary constructs are known to be important barriers to healthy eating as they often concern the rejection of healthy food items, such as fruits and vegetables, partly based on the preference for certain perceptual properties of food, such as visually perceived texture [22,23].

Therefore, the aim of this explorative study is twofold: (i) to investigate the link between children’s general food texture preferences and their sensory sensitivity and neophobic levels; and (ii) to explore whether other factors such as parental texture preferences are correlated with those of their children.

## 2. Materials and Methods

### 2.1. Participants

An online survey was completed by a total of 140 participants: 70 children aged 6–13, together with one of their parents (70 adults). The invitation to participate in the study was forwarded to consumers in the Future Consumer Lab database and the link to the questionnaire was advertised in relevant social media groups. The area of the invitation was limited to Denmark. Parents were thoroughly informed about the project and consented to participate. A voucher worth DKK 50 was used as a financial incentive. This type of research does not require formal ethical approval in Denmark.

### 2.2. Procedure

The study consisted of an online questionnaire, which was organized in two different sections: The first part (Parental Questionnaire) was filled in exclusively by the mother, father or other responsible caregiver (at the discretion of the family), the second one (Children Questionnaire) was filled in by the child. If children had trouble completing the questionnaires, parents were instructed to read the instructions and help them entering their answer. As the study was conducted in Denmark, questionnaires were in Danish. Participants completed the survey using their own electronic devices.

### 2.3. Measures

#### 2.3.1. Demographic Variables and General Information about the Child

Parents were asked to report their country of residence, their weight, height, age, and device type. They also reported their children’s gender, age, weight, height, dental status (completion of the teeth changing phase, use of braces during mealtimes), and weaning time to semi-solid and solid foods (before the age of 4 months, between 4 and 6 months, between 7 and 9 months, later than 9 months, I do not remember at all).

#### 2.3.2. Food Neophobia

Parents completed the reduced version of the Food Neophobia Scale, rating their level of agreement to 6 statements on their children’s behavior and attitudes towards new foods [24,25]. Each statement was measured on a five-point scale ranging from 1 = “strongly disagree” to 5 = “strongly agree”, see Supplementary Material (Table S1). 

#### 2.3.3. Sensory Sensitivity

This study included the Short Sensory Profile, a 38-item assessment in occupational therapy to determine the responses that an individual has to common sensory input. This tool measures sensory processing in seven different domains, including: tactile sensitivity (non-oral), taste/smell sensitivity, movement sensitivity, under responsive/seeks sensation, auditory filtering, low energy/weak, visual/auditory sensitivity. Three subscales of the questionnaire were used to assess parent’s perceptions of child *tactile sensitivity* (e.g., ‘avoids going barefoot, especially in sand or grass’), taste/smell sensitivity (e.g., ‘avoids tastes or food smells that are typically part of a children’s diet’), and visual/auditory sensitivity (e.g., ‘covers eyes, or squints to protect eyes from light’). In addition, a total sensory sensitivity score was computed from the complete questionnaire. Parents responded to the items using a five-point Likert scale from always to never, with higher scores indicating sensory sensitivity and lower scores indicating normal levels of sensory processing [15].

#### 2.3.4. CFTPQ

Both children and parents completed the Child Food Texture Preference Questionnaire (CFTPQ), a tool developed and validated by Laureati et al. (2020) [13]. The questionnaire is based on a forced choice: participants need to indicate the preferred item between 17 pairs of foods differing in their texture. Stimuli include sweet and savory products varying in hardness (e.g., boiled potatoes vs. mashed potatoes) and in particle content (e.g., orange juice with fruit pieces vs. orange juice without fruit pieces). For every pair, the product designation was also written in words (e.g., “yoghurt with fruit pieces”). To decrease order bias, the order of the 17 food items within each pair in the questionnaire was randomized. To increase the standardization of the original protocol, new high-quality pictures of each food item were taken and substituted to the ones from the original questionnaire [13], see Supplementary Materials (Table S2). The area where the photos were taken was covered with black photo paper to obtain a uniform background and photo flash was used to ensure constant lighting conditions in the room. The digital photos were of high-resolution and stored as JPEG format.

#### 2.3.5. Food Frequency Questionnaire

Parents reported their children’s frequency of consumption of the 34 foods included in the CFTPQ. Possible answers for each food item were: “never”, “less than once a month”, “1–3 times a month”, “1–4 times a week”, “everyday or almost everyday”.

### 2.4. Data Analysis

All statistical data analyses were conducted in R, version 3.6.3 (R Core Team, 2020). Effects showing a *p* ≤ 0.05 were considered significant.

#### 2.4.1. Calculation of the CFTPQ Index

As reported by Laureati et al. (2020) [13], an individual CFTPQ index was calculated for each child and parents. The choice of a hard/particulate version of a food pair was scored as +1, the soft/smooth one was scored as 0. The following formula was used:

$$\mathrm{CFTPQ}=\frac{\mathrm{sum}\;\mathrm{of}\;\mathrm{the}\;\mathrm{scores}\;\mathrm{where}\;\mathrm{the}\;\mathrm{hard}\;\mathrm{or}\;\mathrm{particulate}\;\mathrm{food}\;\mathrm{was}\;\mathrm{preferred}}{\mathrm{total}\;\mathrm{number}\;\mathrm{of}\;\mathrm{the}\;\mathrm{particle}\;\mathrm{pairs}}\;\times \;100$$

Based on the distribution frequency of the CFTPQ indexes participants were segmented according to their texture-liker status using the 25th and 75th percentiles as cut-off. Any differences related to age, gender, weight, height, dental status, and weaning practices in the Soft- and Hard-likers children groups were explored through *t*-test. The same background variables were investigated performing one-way ANOVA on the three groups of children using their texture-liker status as grouping variable. If ANOVA showed a significant effect (*p* < 0.05), post hoc Bonferroni test was used.

#### 2.4.2. Food Neophobia and Children’s Texture Preferences

The answers to the items of the reduced Food Neophobia Scale were summed up (after reversing selected scores) to have a food neophobia score ranging from 6 to 30. Higher scores indicate higher levels of food neophobia. The reliability of the FNS was investigated by calculating internal consistency (Cronbach’s α). The correlation between the CFTPQ index and food neophobia was investigated through Pearson’s correlation.

#### 2.4.3. Sensory Sensitivity and Children’s Texture Preferences

The answers to the items of the Sensory Sensitivity Scale were summed up to have a score ranging from 16 to 80. A higher score indicates a higher level of sensory processing. The correlation between the CFTPQ index and sensory sensitivity score was investigated through Pearson’s correlation. The same variable was investigated performing one-way ANOVA and Bonferroni post hoc test on three groups of children using their texture-liker status as grouping variable.

#### 2.4.4. Correlation between Sensory Sensitivity and Food Neophobia

The correlation between sensory sensitivity and food neophobia was investigated through Pearson’s correlation.

#### 2.4.5. Children and Parental Texture-Liker Status

Two-tailed unpaired comparison between parents’ and children’s CFTPQ scores was performed. The correlation between parental and child CFTPQ indices was investigated through Pearson’s correlation.

#### 2.4.6. Food Consumption Frequency and Children’s Texture Preferences

The consumption frequency of the food items was converted to Daily Frequency Equivalents (DFE) and scores were calculated as follows: DFE of 0 = never, DFE of 0.02 = less than once a month, DFE of 0.07 = 1–3 times a month, DFE of 0.28 = 1–4 times a week, DFE of 1.75 = every day or multiple times a day [26,27]. The average frequency of consumption was calculated separately for solid/particulate and soft/non particulate foods. Then, the correlation between the CFTPQ index and food consumption frequency was investigated through Pearson’s correlation.

## 3. Results

### 3.1. Characteristics of the Population

The online survey was completed by to a total of 140 participants: 70 children aged 6–13 years old, alongside one of their parents (59 mothers and 11 fathers). Children were classified into four age groups: 6–7 years old children, n = 12; 8–9 years old children, n = 26; 10–11 years old children, n = 21; 12–13 years old children, n = 11.

The children population consisted of 58.6% boys and 41.4% girls, whereas mothers comprised 84.3% of the total parental respondents. More detailed characteristics of both populations are reported in Table 1. Most children (78.6%) were breastfed (see Table 1). With respect to weaning practices, 57.1% of children were introduced to semi-solid foods into their diet at 4–6 months; 31.4% at 7–9 months; 2.9% did so before 4 months and 4.3% after 9 months. The percentage of children having one or more loose teeth was 32.9%. Only a minority of children wore a dental brace (4.3%).

### 3.2. Segmentation of Participants

To segment participants according to their texture-liker status, the distribution of the CFTPQ index was calculated. Shapiro-Wilk test revealed that both parental and children’s distributions were normal (children’s CFTPQ distribution: W = 0.97, with *p*-value = 0.07; parental CFTPQ distribution: W = 0.97, *p* = 0.08). As described by Laureati et al. (2020) [13], children with CFTPQ score below the 25th percentile were identified as Soft-likers and comprised 31.4% of the total distribution. On the other hand, 35.7% children had CFTPQ score above the 75th percentile and were classified as Hard-likers. Children with a CFTPQ score between the 25th and 75th percentile were referred to as Undefined texture-likers. The same classification was performed on parental CFTPQ distributions, which resulted in 28.6% adults identified as Soft-likers and 35.7% as Hard-likers. The calculated mean of children’s CFTPQ scores was 52.4 ± 16.1, the parental one was 68.3 ± 13.1.

No significant difference of the average age, gender, weight, height, introduction of solid foods, number of missing teeth and usage of dental braces were found between Soft- nor Hard-likers when compared to the total children population.

Analysis of variance performed on the three groups of children grouped based on their texture-liker status confirmed there were no significant differences on their background variables.

### 3.3. Correlation between Children’s Texture Preferences and Food Neophobia

FNS scores across all children varied from 6 to 23, with a mean score of 16.8 (SD = ± 2.7). Total FNS internal consistency (Cronbach’s α), was 0.80 (N = 70). The correlation between food neophobia and the CFTPQ index investigated with Pearson’s correlation was modestly negative and significant (N = 70, r = −0.26, *p* = 0.027), indicating that neophobic children tended to prefer softer and non-particulate textures.

### 3.4. Correlation between Children’s Texture Preferences and Sensory Sensitivity

Sensory sensitivity scores across all children varied from 16 to 57, with a mean score of 30.9 (SD = ±10.2). The correlation between total sensory sensitivity and the CFTPQ index investigated with Pearson’s correlation was negative and significant (n = 70, r = −0.41, *p* < 0.001), indicating that more sensory sensitive children tended to prefer softer and non-particulate textures. Specifically, a negative and significant correlation was found between the CFTPQ index and every sensory domain measured by the SPP, namely: smell/taste sensitivity (r = −0.40; *p* < 0.001), visual/audio sensitivity (r= −0.33; *p* = 0.005) and tactile sensitivity (r = −0.28; *p* = 0.02).

**Figure 1:**
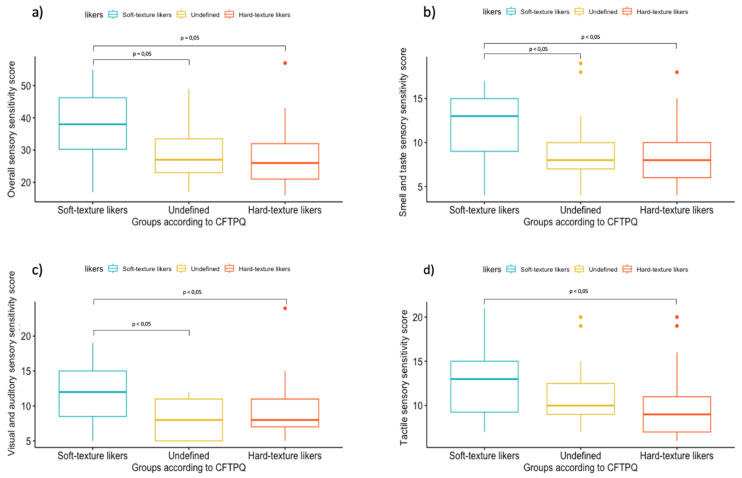
Analysis of variance results in a boxplot form on each Food Texture preference classification resulting for: (**a**) overall sensory sensitivity, (**b**) smell and taste sensory sensitivity, (**c**) visual–auditory sensory sensitivity, and (**d**) tactile sensory sensitivity. The individual colored dots represent outlier values.

Analysis of variance (ANOVA) followed by Bonferroni post hoc test revealed a significant difference between sensory sensitivity scores in the three groups of children (Soft-, Hard- and Undefined-texture likers) (*p* = 0.005) (Figure 1).

### 3.5. Correlation between Sensory Sensitivity and Food Neophobia

The correlation between food neophobia and overall sensory sensitivity investigated with Pearson’s correlation was positive and significant (N = 70, r = 0.47, *p* < 0.001), indicating that children with higher sensitivity to sensory processing also had higher neophobia scores.

### 3.6. Children’s and Parental Texture Preferences

A two-tailed unpaired comparison between parental and children’s CFTPQ scores showed they were significantly different (*p* < 0.001). The parental mean index (M = 68.3) was considerably and significantly higher (*p* < 0.001) than the children mean index (M = 52.4), indicating that parents generally prefer harder textures compared to children.

Pearson’s correlation between child and parental CFTPQ indexes revealed an almost null and non- significant (N = 70, r = 0.04, *p* = 0.72) association. Children’s and parents’ texture preferences matched only in 20% of cases (six matching pairs in the case of Hard-likers and eight in the case of Soft-likers).

### 3.7. Correlation between Children’s Texture Preferences and Food Consumption Frequency

There was a significant and modestly negative correlation between CFTPQ index and frequency of consumption of both soft and non-particulate foods (r = −0.27, *p* < 0.0001), and hard and particulate foods (r= −0.40, *p* < 0.001).

## 4. Discussion

The CFTPQ tool reveals segments of children with different texture preferences (Hard- vs. Soft-likers), as indicated by Laureati et al. (2020) [13]. These groups also differ in their levels of food neophobia and sensory information processing evaluated through parental reports.

### 4.1. Children’s food Texture Preferences

This study revealed a significant difference in how children differently distribute according to their texture-liker status. Specifically, results showed a higher percentage of Danish children preferring more complex textures (i.e., hard and particulate), in line with a recent cross-sectional study showing children from Northern countries (such as Sweden) being more frequently classified as Hard-texture likers when compared to children from Southern countries (such as Spain) [13]. Arguably these similarities in Northern countries may be explained by affinity in preparation methods and culinary habits, considering that the ranges of food a child is exposed and hence develop preferences towards are greatly determined by culinary traditions [28,29]. Previous research has also underlined the importance of age in the development of varied food texture preferences in children in the 7–10 years old range [7,11]; therefore, an age effect between Soft- and Hard-likers was expected. Surprisingly, our study did not find any age differences between the two groups, which were also comparable for all other background variables investigated, namely gender, BMI, use of braces, and weaning practices; consistently with a previous study [13]. However, it cannot be ruled out that our sample size was too small, so further research should confirm and extend our findings by including a greater number of children, especially in the extreme age range, namely the 6 to 7 years old range (which only comprised 12 children) and the 12–13 years old range (which only included 11 children).

### 4.2. Correlation between Texture Preferences, Children’s Sensory Processing and Food Neophobia

One of the most interesting results of the present study is the association between texture preference and children’s sensory processing, in the domain of smell/taste, visual/audio, and tactile sensitivity. The latter can be conceptualized both as having better tactile perception and as stronger affective response to tactile stimulation [30]. Some studies have supported the assumption that higher sensitivity to touch contributes to picky eating. For instance, a group of children clinically diagnosed with tactile defensiveness (children who display an overreaction to tactile stimulations) rejected more foods, ate fewer vegetables and refused more often to eat new foods compared to healthy children [31]. Our findings make a step forward and point out the possibility that children being more sensitive to all kinds of sensory stimulation (including not only touch and taste but extending to sounds, light, and smells) may have specific preferences for softer and more uniform textures. This is particularly important given the interplay between sensory sensitivity, food neophobia and the rejection of foods underlined by previous research both in young children and in adult picky eaters [17,32,33]. In our study, Hard-likers were found to be more neophilic and less sensory sensitive, showing an inclination towards harder and particulate foods [13,34]. On the other hand, Soft-likers’ rejection of more complex textures was found to be associated with selective eating behavior and a higher degree of sensory processing, consistently with previous research [11,13,32,35,36].

### 4.3. Correlation between Child and Parental Texture Preferences

The second aim of this study was to investigate whether an association between children’s and parents’ texture preferences exists. Despite the lack of age-effect within the pediatric population, the comparison between children’s and parental CFTPQ scores clearly indicated a preference shift towards more complex textures during adulthood; consistently with previous studies [11,13,31]. Indeed, adults preferred foods that were harder and contained particles when compared to their children. This may be due to physiological roots: the progressive development and change in masticatory functions (chewing and swallowing) across the lifespan could allow to handle complex textures more easily, that in turn could be more accepted and preferred [11,37]. In addition, these age-related differences in texture preferences could also be due to a greater exposure to a wider variety of foods with harder and lumpier textures, that would result in a progressive shift of preference in adulthood. Very few studies have investigated whether such different texture preferences could be ascribed to perceptual differences in lingual tactile acuity, but adults and children have been shown to have comparable performances [11,33]. However, when considering oral sensitivity to specific texture dimensions (such as flowability) using real food products, different degrees of sensitivity have been shown to drive food choice and preference in adults [38]. 

Moreover, despite our study not revealing any gender effect on children’s and adults’ texture preferences, recent works suggest that some gender-specific trends may exist for the sensitivity to specific texture attributes, such as graininess and hardness [39,40]. It would be interesting to investigate both these aspects among the pediatric population and to determine whether a familial resemblance between children and their parents exists. 

Finally, it has been shown that parental levels of food neophobia, as well as of other heritable traits (including tactile sensory processing) are strongly correlated with those of their children’s, possibly because of the reduced exposure to food and sensory stimuli that parents themselves find distressing [19,33,41,42]. Interestingly, this resemblance appears to be in contrast with the lack of alignment regarding children’s and parental texture preferences we found in our study. Future studies may look at other possible ways in which children’s texture preferences are developed and shaped.

### 4.4. Strengths and Limitations

A strength of our study is that the CFTPQ tool was used to investigate children’s texture preferences on a wide age-range allowing to underline some associations with general sensory sensitivity; a topic which has not been widely explored.

However, we acknowledge that our results are based on parental reports, and therefore a behavioral measure to evaluate sensory processing in differing domains and its influence on texture preferences development would be warranted to confirm and generalize our findings. Additionally, it must be noticed that our study is based on visual stimuli only, and therefore a reproduction of this design with tangible products in the future would allow for a comparison between self-reported visual preference and actual food choices.

When investigating children’s individual characteristics affecting texture preferences, this study suggests placing greater emphasis on the role of general sensory sensitivity. Additionally, future studies could examine the efficacy of exposure techniques to increase acceptance of harder and lumpier food textures in children who are over-responsive to sensory information.

Furthermore, even though we did not identify any associations between parents’ and children’s texture preferences, it would be interesting to investigate other ways in which parents may influence children’s texture preferences, such as through their parenting style.

## 5. Conclusions

This study was conducted to better investigate the role of sensory sensitivity and food neophobia on children’s food texture preferences and to explore whether other variables such as parental texture preferences relate to their children’s.

The results showed that children who differed in their texture-liker status also differed in their levels of food neophobia and sensory information processing. No relationship was found between parental and children’s texture preferences, but further studies are needed to determine the extent to which different texture preferences across the lifetime are based on experience, physiological and/or perceptual factors.

## Figures and Tables

**Table 1:** Characteristics of the participants.

	Variables	
**Children**	*N* *Gender (% girls)* *Age in years (mean; SD)* *Weight in kg (mean; SD)* *Height in cm (mean; SD)* *Breastfeed (% yes)* *Missing teeth (% yes)* *Dental brace (% yes)*	70 41.4 9.4 ± 1.9 33.9 ± 10.1 138.9 ± 78.6% 34.3% 4.3%
**Adults**	*N* *Gender (% females)* *Age in years (mean; SD)* *Weight in kg (mean; SD)* *Height in cm (mean; SD)*	70 84.3% mothers 40.7 ± 4.2 72.0 ± 13.9 170.8 ± 8.0

## Data Availability

Data is not available for sharing.

## Supplementary Materials

The following are available online at https://www.mdpi.com/article/10.3390/foods10102327/s1, Table S1: Danish version of the reduced Food Neophobia Scale. Table S2: The Child Food Texture Preference Questionnaire (Danish and English version).
