# Supplementary material for: Food Texture Acceptance, Sensory Sensitivity, and Food Neophobia in Children and Their Parents
Source: Foods. 2021 Sep 30;10(10):2327. doi: 10.3390/foods10102327 (PMC8535628; doi:10.3390/foods10102327)
Supplement: Supplementary file 1 [file foods-10-02327-s001.zip › foods-1368702-supplementary.pdf]

Supplementary materials Table S1: Reduced Food Neophobia Scale provided to parents

The English version of the Reduced Food Neophobia Scale can be found in black text and the Danish translation in blue coloured text.

	Strongly disagree	Disagree	Neutral	Agree	Strongly agree
	Meget uenig	Uenig	Neutral	Enig	Meget enig
My child is constantly sampling new and different foods.<br>Mit barn prøver konstant nye og forskellige fødevarer.					
My child doesn't trust new foods.<br>Mit barn stoler ikke på nye fødevarer.					
If my child doesn't know what is in a food, he/she won't try it.<br>Hvis mit barn ikke ved, hvad der er i maden, vil han/hun ikke prøve at smage den.					
My child is afraid to eat things he/she has never had before.<br>Mit barn er bange for at spise ting, han/hun aldrig har fået før.					
My child is very particular about the foods he/she will eat.<br>Mit barn er meget bestemt omkring de fødevarer, han/hun vil spise.					
My child eats almost anything.<br>Mit barn spiser næsten hvad som helst.					

Supplementary materials Table S2: The Child Food Texture Preference Questionnaire

The English version of the CFTPQ can be found in black text and the Danish translation in blue text.

Which product do you prefer?

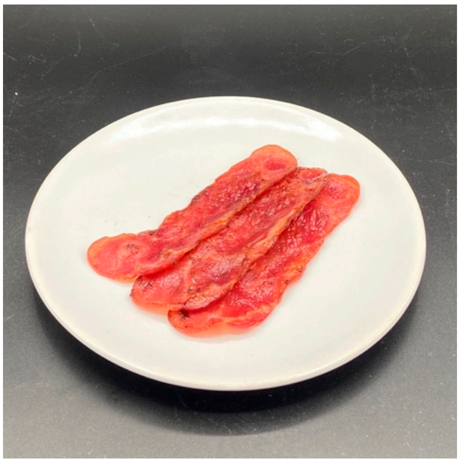

Crispy turkey bacon - Sprød kalkunbacon

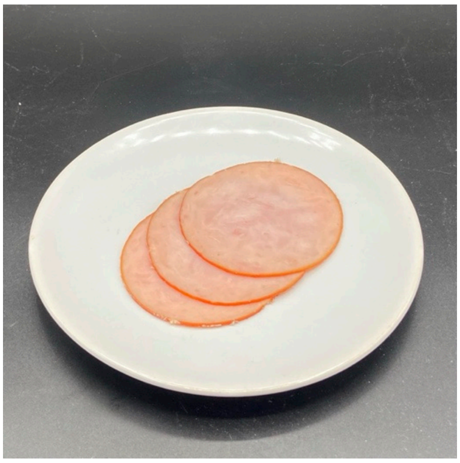

Turkey slices - Kalkunskiver

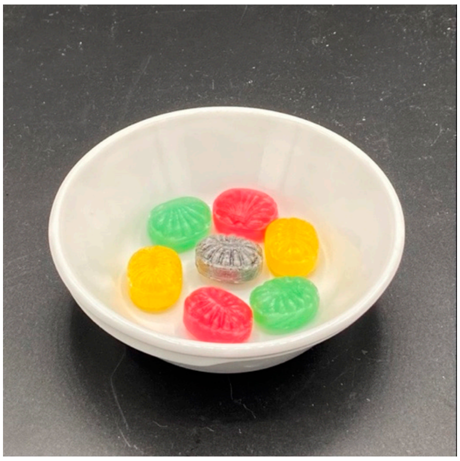

Hard candy - Bolscher

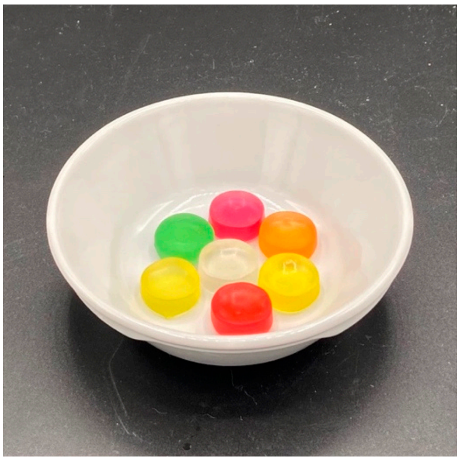

Winegums - Vingummi

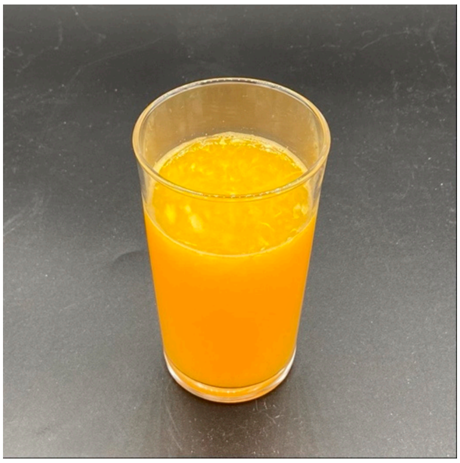

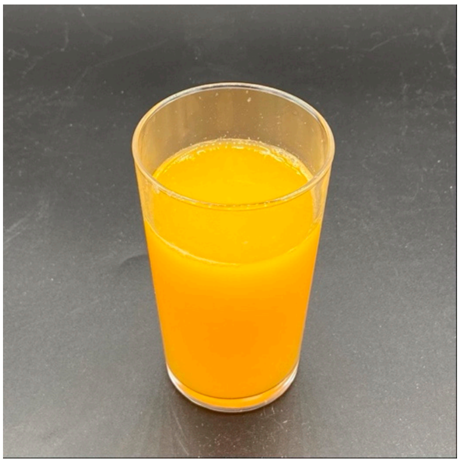

Orange juice with pulp –
Appelsinjuice med frugtkød

Orange juice without pulp -
Appelsinjuice uden frugtkød

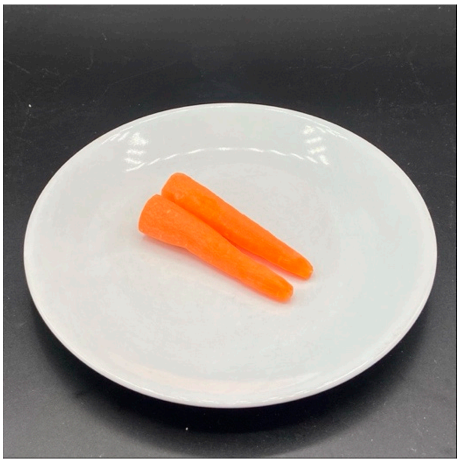
Raw carrots - Rå gulerod

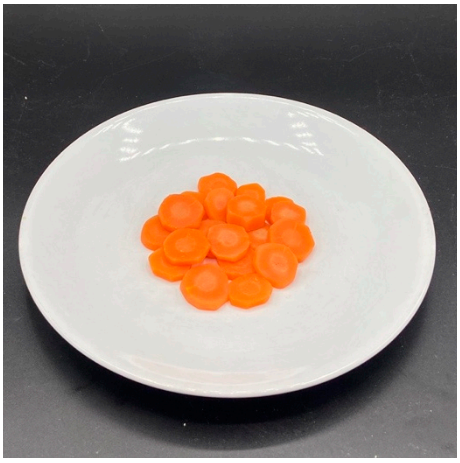
Boiled carrots - Kogt gulerod

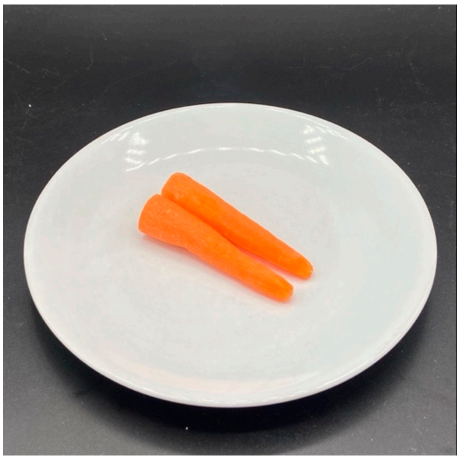
Raw carrots - Rå gulerod

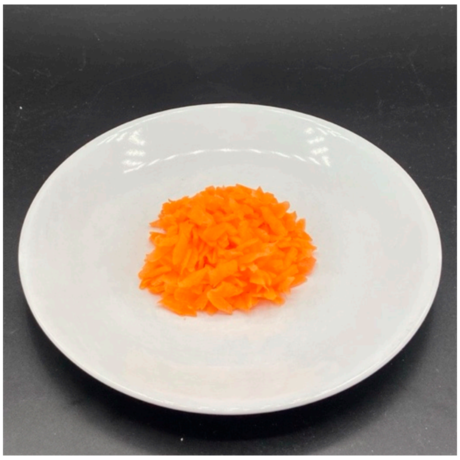
Raw grated carrots - Revet gulerod

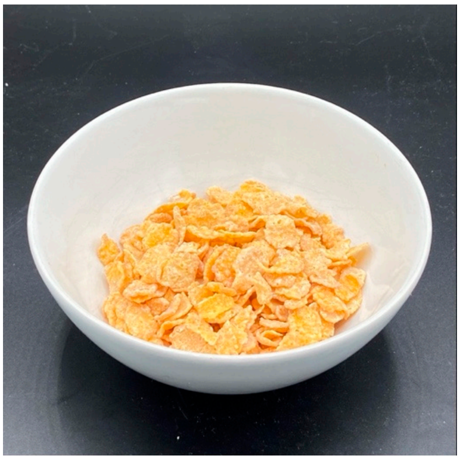
Crispy corn flakes - Sprøde cornflakes

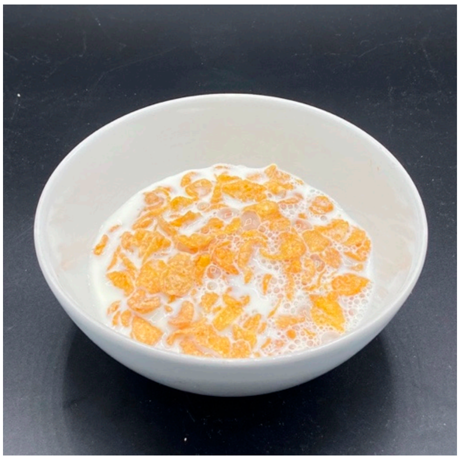
Softened corn flakes - Blødgjorte cornflakes

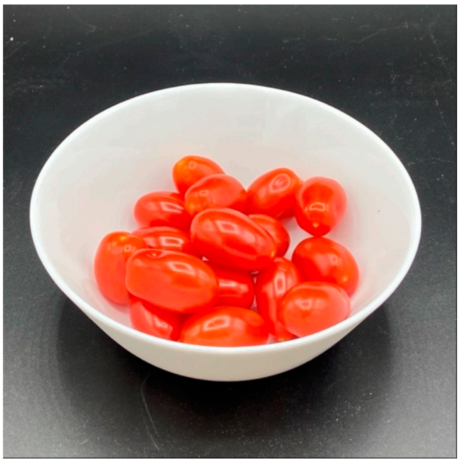
Cherry tomatoes - Cherry-tomater

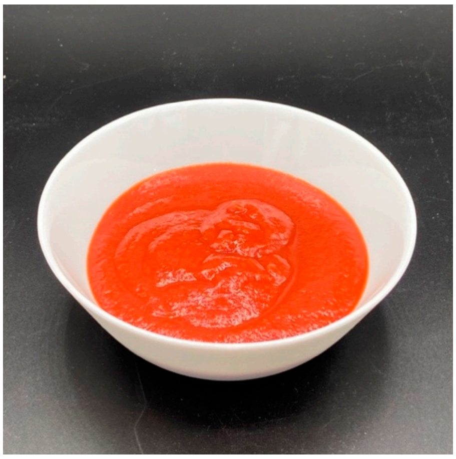
Tomato soup - Tomatsuppe

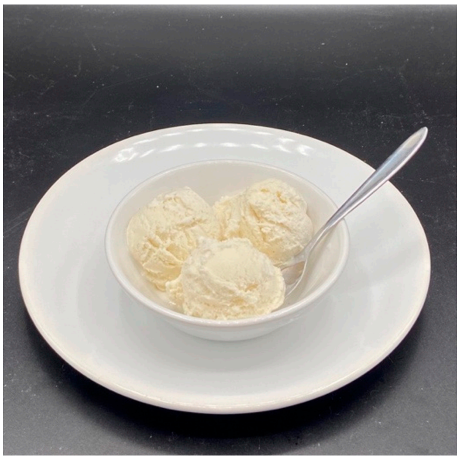
Hard ice cream - Hård is

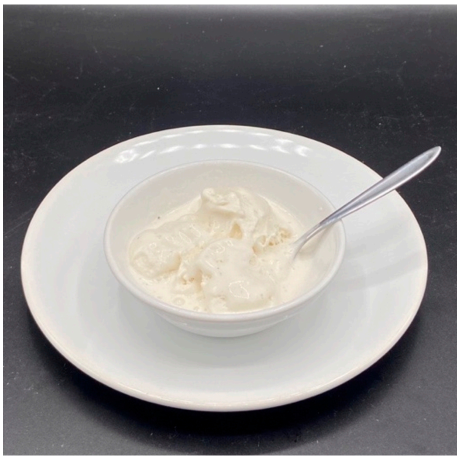
Soft-serve ice cream - Softice

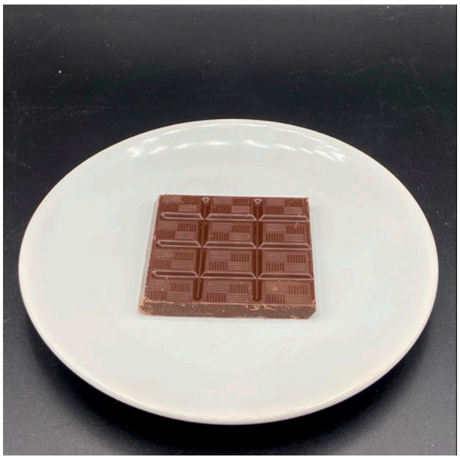
Chocolate bar - Chokoladebar

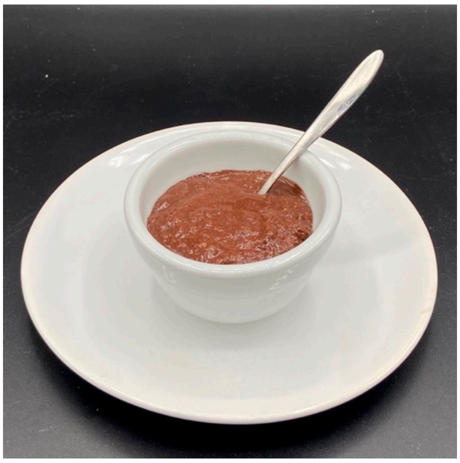
Chocolate mousse - Chokolademousse

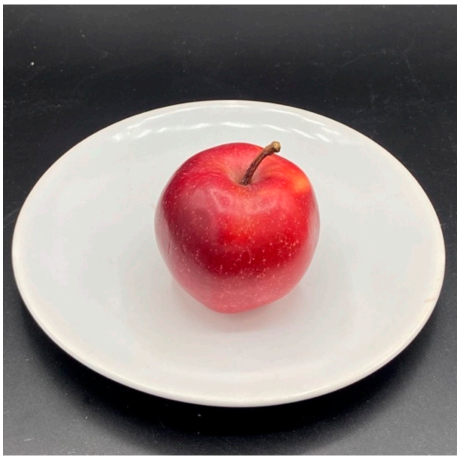
Apple - Æble

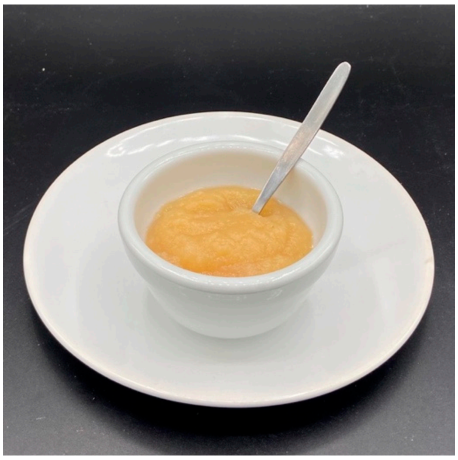
Apple puree - Æblepuré

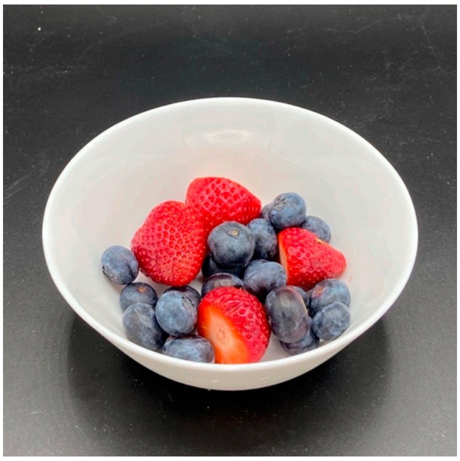
Berries - Bær

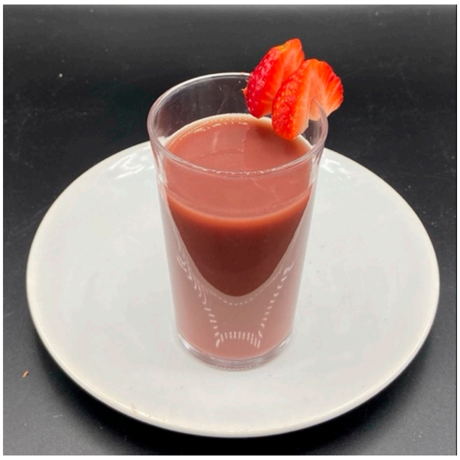
Berry smoothie - Bærsmoothie

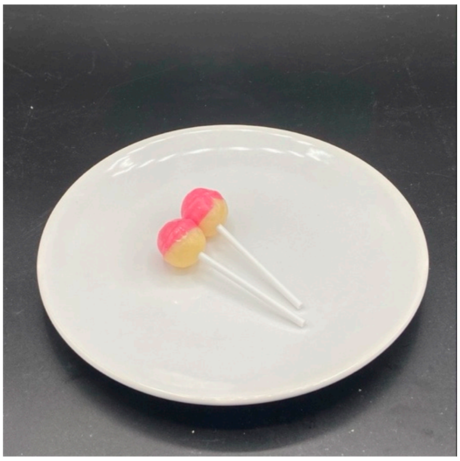
Lollipop - Slikkepind

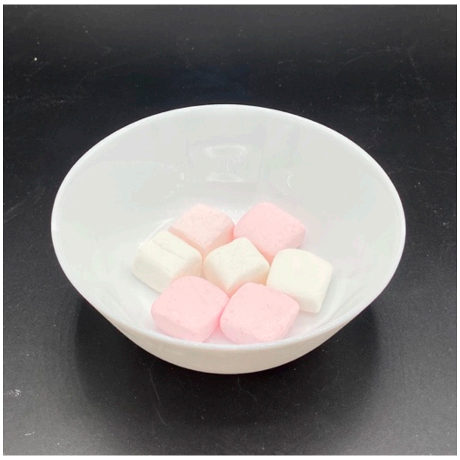
Marshmallows - Skumfiduser

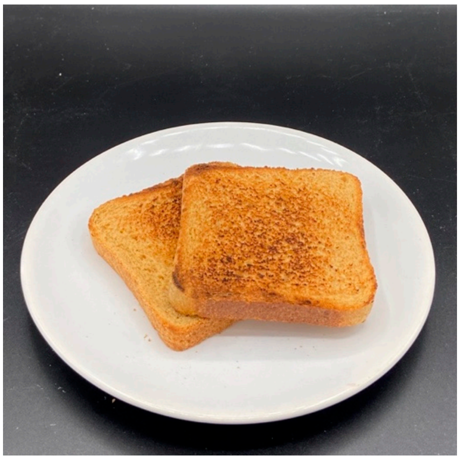

Toasted bread – Ristet brød

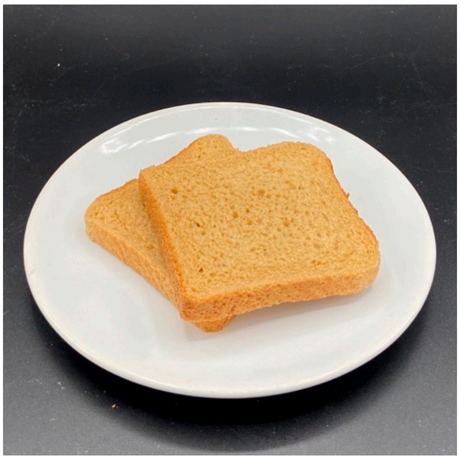

Bread - Brød

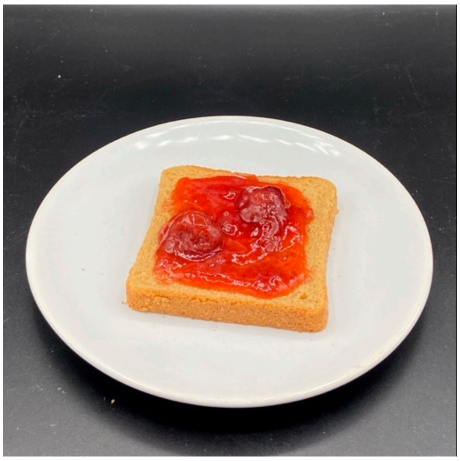

Jam with fruit pieces - Syltetøj med frugtstykker

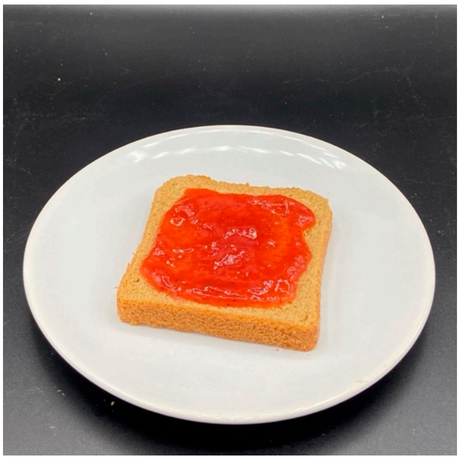

Jam without fruit pieces - Syltetøj uden frugtstykker

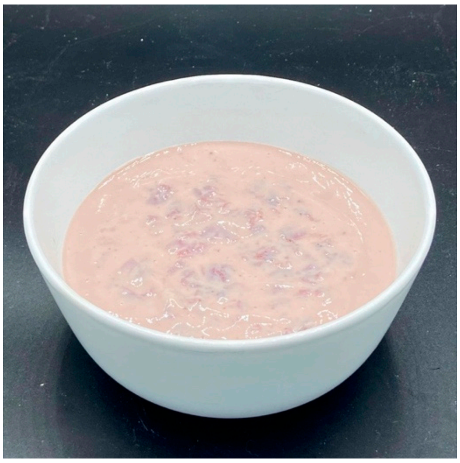

Yoghurt with fruit pieces - Yoghurt med frugtstykker

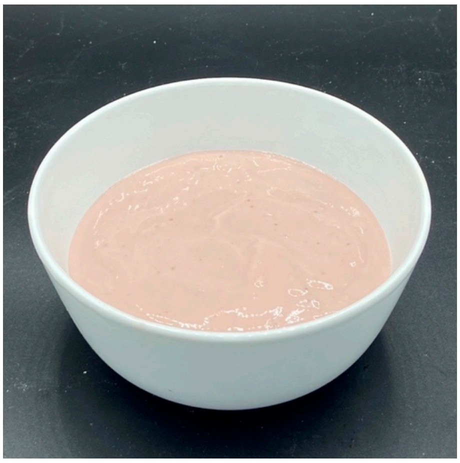

Yoghurt without fruit pieces - Yoghurt uden frugtstykker

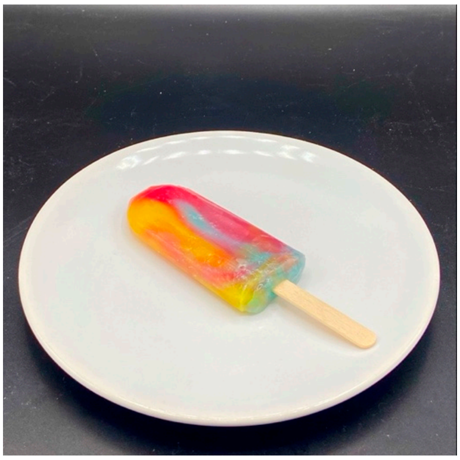

Ice stick - Ispind

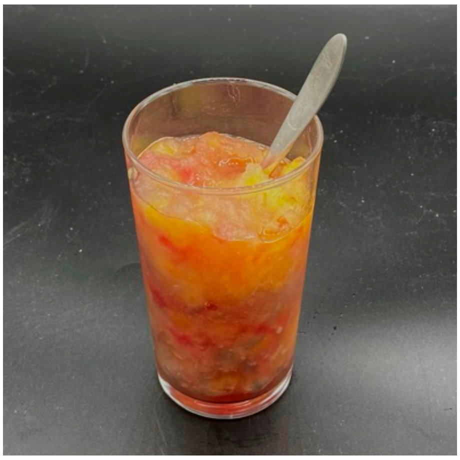

Slush ice - Slush ice

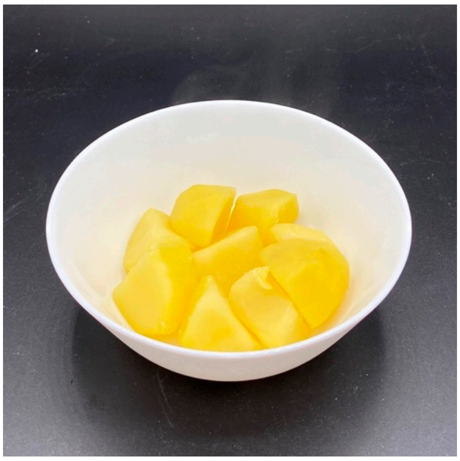

Boiled potatoes - Kogte kartofler

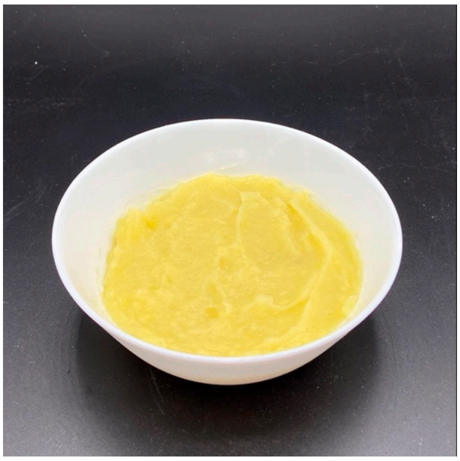

Mashed potatoes - Kartoffelmos
